# Intestinal microbiota and its interaction to intestinal health in nursery pigs

**DOI:** 10.1016/j.aninu.2021.05.001

**Published:** 2021-06-12

**Authors:** Marcos Elias Duarte, Sung Woo Kim

**Affiliations:** Department of Animal Science, North Carolina State University, Raleigh, NC, 27695, United States

**Keywords:** Intestinal health, Mucosa-associated microbiota, Nursery pig

## Abstract

The intestinal microbiota has gained increased attention from researchers within the swine industry due to its role in promoting intestinal maturation, immune system modulation, and consequently the enhancement of the health and growth performance of the host. This review aimed to provide updated scientific information on the interaction among intestinal microbiota, dietary components, and intestinal health of pigs. The small intestine is a key site to evaluate the interaction of the microbiota, diet, and host because it is the main site for digestion and absorption of nutrients and plays an important role within the immune system. The diet and its associated components such as feed additives are the main factors affecting the microbial composition and is central in stimulating a beneficial population of microbiota. The microbiota–host interaction modulates the immune system, and, concurrently, the immune system helps to modulate the microbiota composition. The direct interaction between the microbiota and the host is an indication that the mucosa-associated microbiota can be more effective in evaluating its effect on health parameters. It was demonstrated that the mucosa-associated microbiota should be evaluated when analyzing the interaction among diets, microbiota, and health. In addition, supplementation of feed additives aimed to promote the intestinal health of pigs should consider their roles in the modulation of mucosa-associated microbiota as biomarkers to predict the response of growth performance to dietary interventions.

## 1 Introduction

The interaction between intestinal health and microbiota on the growth of pigs has received increased attention by the swine industry and academia (Kim and Duarte, 2021). Modulation of intestinal microbiota can lead to immediate and long-term effects on the intestinal health of pigs (Jang et al., 2020a; Schokker et al., 2015). The establishment of robust microbiota in the early life of pigs is extremely important for growth of pigs as it is related to the development of intestinal functions and immune system (Chen et al., 2018c; Kabat et al., 2014; Li et al., 2018a). Feeds and their associated nutritional components are the major factor affecting the microbiota profile in the intestine of pigs (Wang et al., 2019a). Consequently, the modulation of the intestinal microbiota can be key to the development of strategies to promote enhanced responses to nutritional interventions.

The intestine of pigs is colonized by a dense, dynamic, and highly complex community of microorganisms composed mainly of bacteria (Isaacson and Kim, 2012). Along the intestine and from the mucosa to the lumen, the microbiota pattern markedly changes due to the physicochemical differences in the microenvironment (Adhikari et al., 2019; Crespo-Piazuelo et al., 2018; Gresse et al., 2019). The mucosa-associated microbiota directly interacts with intestinal immune cells increasing their capacity to modulate the immune system (Arpaia et al., 2013; Belkaid and Hand, 2014; Mulder et al., 2011). In addition, the majority of cells from the immune system are located in the intestine (Mowat and Agace, 2014).

Mucosa-associated microbiota act as the frontline defenders against pathogens by competitive exclusion and immune status modulation (Belkaid and Hand, 2014; Brandtzaeg, 2007; Ma et al., 2018). Production of immunoglobulin A (IgA) induced by microbiota modulates bacterial colonization, preventing the translocation of bacteria through the epithelial layer (Gutzeit et al., 2014). Moreover, microbiota could metabolizes certain toxins from feeds and can synthesize certain vitamins that can be used by intestinal epithelium of the host (Yang et al., 2016). Furthermore, the intestinal microbiota are shown to support the maturation of the intestinal epithelial cells and their barrier functions, promoting homeostasis of the intestinal immune system (Kabat et al., 2014; Li et al., 2018a; de Vries and Smidt, 2020).

Therefore, this review is focused on the role of mucosa-associated microbiota in the intestinal health of pigs and the interaction among the diets, microbiota, and immune system in the intestine of pigs.

## 2 Establishment and development of intestinal microbiota

Early establishment of the intestinal microbiota is extremely important for the maturation of the intestinal immune system, barrier function, and consequently the health and growth of pigs (Kabat et al., 2014; Li et al., 2018a; de Vries and Smidt, 2020). In addition, intestinal microbiota have also been related to promoting growth in neonates by increasing the sensitivity to growth hormone (Shanahan et al., 2017). Development of intestinal microbiota is related to factors including host genome, breed age, sex, and diets (Adhikari et al., 2019; Bergamaschi et al., 2020; Crespo-Piazuelo et al., 2019; Verschuren et al., 2018). Furthermore, early interventions on the establishment and development of the intestinal microbiota has been shown to induce long-lasting effects (Everaert et al., 2017; Schokker et al., 2018).

### 2.1 Early establishment of intestinal microbiota

Whether the intestine is first exposed to microbials in the uterus or during birth is still controversial (Ardissone et al., 2014; de Goffau et al., 2021; Rackaityte et al., 2021; Stinson et al., 2019). The possible intrauterine microbiota colonization or fetal exposure to microbiota metabolites from the maternal gut microbiota would have a significant effect on the development of intestinal functions and the immune system altering postpartum colonization (Nowland et al., 2019). Wang et al. (2019a) evaluated the development of intestinal microbiota in pigs and reported that microbiota in meconium samples differed from microbiota collected from fecal samples during lactation. Although the meconium samples were collected within 6 h after birth, the authors suggested that the meconium microbiota could have been transmitted from the sow in-utero. However, the most accepted concept is that the neonates have the microbiota initially established during parturition.

### 2.2 Postnatal establishment and development of intestinal microbiota

At birth, piglets face with a substantial load of microorganisms from the birth canal and the sow's feces (Nowland et al., 2019). In commercial conditions, suckling piglets are housed in the same crate with the mother and have contact with feces, mucosal surfaces, skin, and fluids until weaning (Nowland et al., 2019). Moreover, it has been demonstrated that the vaginal microbiota plays an important role in the early colonization of the intestinal microbiota of the offspring (Pena Cortes et al., 2018) with the vaginal microbiota also being influenced by the feces of the sow (Chen et al., 2018c). Consequently, the sow fecal microbiota greatly contribute to the development of the offspring microbiota in the following days after birth (Morissette et al., 2018).

Immediately after birth, piglets start intestinal nutrition by suckling the sow to obtain colostrum and milk. Maternal milk provides energy and nutrients including lactose, milk oligosaccharides, amino acids, and fat (Kim, 2013) that activate digestive functions and in turn alter the environment for intestinal microbiota colonization (Everaert et al., 2017; Liu et al., 2019). According to Wylensek et al. (2020), heavier piglets have been shown to have a greater abundance of Bacteroidetes, *Bacteroides*, and Ruminococcaceae and lower proportions of *Actinobacillus porcinus* and *Lactobacillus amylovorus* compared with lighter piglets. The authors suggested that the quantity of milk ingested during lactation may potentially affect the health and performance of the host through modulation of the intestinal microbiota. The nutritional components of maternal milk include oligosaccharides that contribute greatly to the development of the intestinal microbiota (Salcedo et al., 2016). In addition, Schokker et al. (2018) reported that oral fructooligosaccharide administration to suckling pigs increased the relative abundance of Lactobacillaceae and Bifidobacteriaceae in colonic digesta and enhanced barrier function whereas reducing the expression of cytokine signaling in jejunal mucosa.

Besides the nutrients in colostrum and milk, the bioactive compounds including immunoglobulins, antimicrobial, anti-inflammatory factors, and microbiota also contribute to the intestinal microbiota establishment and development especially in neonatal pigs with an immature immune system (Chen et al., 2018a). Whereas, the composition of colostrum is markedly different from milk (Kim, 2013). The IgA concentration reduces from 21.2 to 6.7 mg/mL 18 h following farrowing (Klobasa et al., 1987). The reduction on the IgA concentration can be related to the variation on the intestinal microbiota during lactation. Immunoglobulin A, the most abundant immunoglobulin in sow colostrum and milk, binds to pathogens impairing their replication (Moor et al., 2017) and helps to prevent bacterial adhesion to intestinal epithelial cells (Dunne-Castagna et al., 2020). According to Wang et al. (2019a), the microbial diversity of pigs reduced drastically on d 11 of lactation and increased on d 20 before weaning. Rogier et al. (2014) reported that the intestinal microbiota of mice that received maternal milk IgA was different at weaning when compared to those that did not receive IgA. This difference was even greater in adult mice, indicating that the milk IgA promotes a long-lasting effect on the intestinal microbiota.

According to Starke et al. (2013) and Paβlack et al. (2015), the nutritional manipulation of the sow intestinal microbiota can affect the luminal microbiota in the intestine of the offspring. Moreover, Baker et al. (2013) reported that the offspring of sows fed a supplemented diet with *Bacillus subtilis* resulted in increased *Lactobacillus* spp. and reduced *Clostridium perfringens* in the ileum. Ma et al. (2020) reported that nursing piglets from sows fed a diet supplemented with a synbiotic had significant changes in the luminal microbiota in colon and further reduced the systemic immune and oxidative stress status. Conversely, considering the sow diet is different from piglets, the shape of the intestinal microbiota alteration in sows differs from their offspring (Leblois et al., 2017). Furthermore, Choudhury et al. (2021) reported that suckling pigs receiving a creep-feed modulated the population of *Ruminococcus, Lachnospira,* Lachnospiraceae*, Roseburia, Papillibacter, Eubacterium,* and *Prevotella* in colonic digesta which was associated with their intestinal development at weaning. These results indicate that the microbiota can be manipulated in early life inducing long-lasting effects. The modulation of the piglet microbiota greatly depends on the environment and can start during gestation and lactation by modulating the sow's intestinal microbiota and milk composition.

### 2.3 Development of microbiota after weaning

At weaning, pigs face nutritional, environmental, physiological, and psychological challenges causing weaning stress (Campbell et al., 2013; Montagne et al., 2007). Weaning stress may lead to a disruption or dysbiosis in the intestinal microbiota, the major factor contributing to post-weaning infections (Gresse et al., 2017; Konstantinov et al., 2006). Li et al. (2018b) reported that weaning increased mainly Lachnospiraceae, Negativicutes, Selenomonadales*,* Campylobacterales, whereas decreased *Campylobacter*, Porphyromonadaceae*, Alloprevotella, Barnesiella,* and *Oscillibacter*. Moreover, after weaning the relative abundance of *Prevotella* increases in weaned piglets with introduction of a plant-based diet (Guevarra et al., 2018). The diet being fed and ingested is the major factor in modulation of the intestinal microbiota (Bian et al., 2016; Frese et al., 2015; Niu et al., 2015; Tilocca et al., 2017; Wang et al., 2019a). The shift in the intestinal microbiota profile is attributed mainly to the abrupt transition from liquid milk to a solid plant-based diet that affects the physicochemical conditions and the substrate availability in the intestine (Bian et al., 2016), in addition to the reduction in the immunoglobulin supply from milk (Dunne-Castagna et al., 2020). The alteration of the intestinal microbiota due to weaning stress also changes the bioactive compounds (Li et al., 2018a) and the expression of genes related to nutrient metabolism (Meng et al., 2020). Therefore, psychological stress caused by weaning may play a role in the intestinal microbiota. Galley et al. (2015) reported the stressors caused changes in both the luminal and mucosa-associated microbiota in the colon of murine. In light of this, nutritional strategies have been implemented in an attempt to stimulate beneficial microbiota proliferation while providing an environment that is detrimental to pathogens. Lo Verso et al. (2020) evaluated a blend of additives composed of bovine colostrum, cranberry extract, carvacrol, yeast-derived mannans, and β-glucans and reported an increase in the abundance of beneficial bacteria such as *Lactobacillus reuteri* and *Faecalibacterium prausnitzii* and reduced the abundance of *Helicobacter* in ileal mucosa enhancing the systemic health status and growth performance of nursery pigs. The authors correlated these results due to the complementary functional properties of the additives within the blend.

Wang et al. (2019a) evaluated the dissimilarity in intestinal microbiota of pigs at different growth stages. The authors concluded that the microbiota of piglets from lactation is distinct from pigs at the nursery phase with *R* = 0.98. Whereas the microbiota from the nursery, growing, and finishing pigs were more similar to each other with *R* ranging from 0.43 to 0.55. These results showed that after recovery from weaning stress the microbiota shifts toward maturation. The smaller differences among plant-based diet phases could be because of the similarity of the basal diets after weaning. Indeed, after colonization, some microbes persist in the intestine from lactation to the finishing phases (Wang et al., 2019a). The stability of the intestinal microbiota indicates gut microbiome maturity. According to Ke et al. (2019), maturation of the intestinal microbiota normally occurs around 80 d of age in pigs, whereas Zhao et al. (2015) indicated that the intestinal microbiota are relatively stable at 6 months of age. It is ambiguous to determine when intestinal microbiota mature because intestinal microbiota is dynamically affected by several factors including diets and the host immune system maturation. Therefore, it can be suggested that the maturation of intestinal microbiota occur during early life from weaning when pigs receive plant-basal diets to finishing phase which is also related with the maturation of immune system (Honda and Littman, 2016).

## 3 Composition of intestinal microbiota

The physicochemical conditions and substrate availability constantly change along the gastrointestinal tract modulating the microbiota toward a different pattern (Adhikari et al., 2019; Crespo-Piazuelo et al., 2018; Gresse et al., 2019; Zhao et al., 2015). According to Zhao et al. (2015), the similarity of the microbiota in the feces is 0.75 and 0.38 compared with the luminal microbiota of the large intestine and small intestine, respectively.

The large intestine is the major place for microbial fermentation in pigs with greater microbial diversity compared with the small intestine (Adhikari et al., 2019; Kelly et al., 2017). In addition, the microbiota in the lumen of large intestine play an important role in degrading fiber and in energetic metabolism (Den Besten et al., 2013; Niu et al., 2015). However, the jejunum is a major site for digestion and absorption of nutrients and a significant amount of fiber fermentation still occurs in the small intestine (Chen et al., 2020; Passos et al., 2015). Crespo-Piazuelo et al. (2018) reported that the greater nutritional function of the microbiota in the jejunum is more related to the energetic metabolism and fiber degradation. In addition to digestive functions, the intestinal microbiota produce bioactive compounds that can affect the jejunal immune system, barrier function, and cell proliferation (Jin et al., 2020). According to Zhao et al. (2015), the microbiota of the small intestine contained more immune functions related to disease, cancer, and infectious disease compared with that from the large intestine. Additionally, Wiarda et al. (2020) reported that T cell populations are more abundant in the small intestine than in the large intestine in pigs at 8 wk of age, whereas it was similar in pigs at 4 wk of age. Petry et al. (2021) reported that the interaction among diet, microbiota, and immune system responses is more effective in the small intestine. Furthermore, Gresse et al. (2019) suggested that the characterization of fecal microbiota could prove inadequate in investigating post-weaning infections etiology due to intestinal infections or multiplication sites being located in the jejunum, ileum, or the colon segments. Therefore, considering the majority of the dietary compound are digested, absorbed, and metabolized in the small intestine, exposing the mucosa to various exogenous antigens and microbial components from the diet, the jejunum seems to be a key site to analyze the interaction among diets, intestinal microbiota, and intestinal health.

### 3.1 Significance of mucosa-associated microbiota

The majority of the studies focused on evaluating the microbiota in animal models have utilized luminal or fecal samples. However, the microbiota interaction with the host in combination with the physicochemical properties of luminal content leads to a distinct microbiota profile along both the radial and longitudinal axis of the mammalian intestine (Albenberg et al., 2014; Friedman et al., 2018). An increasing number of studies have been done to investigate the interaction of the jejunal mucosa-associated microbiota and diet in pigs (Duarte et al., 2020; Jang et al., 2020a; Kim et al., 2019; Li et al., 2017). Moreover, post-weaning dietary intervention has been shown to have a long-lasting effect on mucosa-associated microbiota but not on digestion in the small intestine (Levesque et al., 2012; Adhikari et al., 2019).

It has been demonstrated that the mucosa-associated microbiota are markedly different from those of the luminal content in pigs (Adhikari et al., 2019; Burrough et al., 2017; Mu et al., 2017; De Rodas et al., 2018). Luminal microbiota interact more with the digesta thus affecting nutrient digestion in addition to secretion of metabolites, whereas the mucosa-associated microbiota are shown to directly crosstalk with intestinal immune cells (Arpaia et al., 2013; Belkaid and Hand, 2014; Mulder et al., 2011) and are more susceptible to dietary influence in the small intestine (Levesque et al., 2012, 2014). Mu et al. (2017) reported that mucosa-associated microbiota may have a greater capability on immunological regulation. Mucosa-associated microbiota have the ability to attach to mucin glycans in the intestinal epithelial cells to further proliferate and interact with the host (Etienne-Mesmin et al., 2019). Moreover, Liu et al. (2019) concluded that evaluating only the fecal microbiota is insufficient to understand the mechanisms of development of the intestinal microbiota and immune system. The interaction between the mucosa and microbiota can effectively modulate the immune system, providing a line of defense for the host by preventing pathogenic colonization (Belkaid and Hand, 2014; Brandtzaeg, 2007; Buffie and Pamer, 2013; Ma et al., 2018; Matsubara et al., 2017; Shi et al., 2017; Stokes, 2017). Furthermore, Yang et al. (2020) concluded that the mucosa-associated microbiota were correlated with diarrhea-predominant irritable bowel syndrome in humans whereas the luminal microbiota did not differ from healthy individuals.

Therefore, changes in mucosa-associated microbiota may have marked effects on the growth and development of the host (Adhikari et al., 2019; Niu et al., 2015; Shi et al., 2017). It is worth mentioning that the different intestinal segments and their niches (mucosa and lumen) are not completely independent. The microbiota can co-inhabit both luminal and mucosa environments (Zhang et al., 2018a). Furthermore, the intestinal microbiota can be affected to different degrees in luminal and mucosal by the same factor (Galley et al., 2015). According to Petry et al. (2021), pigs fed high fiber diet had increased Erysipelotrichaceae*, Olsenella*, and *Turibacter* in the ileal lumen, whereas it increased *Turibacter, Helicobacter,* and Lachnospiraceae in the ileal mucosa. The authors also reported that pigs fed high fiber diet supplemented with xylanase had increased Lachnospiraceae*, Actinobacillus*, *Bifidobacterium*, and *Lactobacillus* and reduced *Streptococcus* and *Turicibacter* in the ileal lumen, whereas it increased *Bifidobacterium*, *Megasphaera*, and *Chlamydia*; and reduced *Clostridium* and *Escherichia*, and *Shigella* in the ileal mucosa. When pigs were fed a high fiber diet, supplemented with arabinoxylan-oligosaccharides (AXOS), the ileal lumen had increased Lachnospiraceae and reduced *Actinobacillus*, whereas the ileal mucosa showed increased *Megasphaera* and *Streptococcus*, and reduced *Candidatus arthromitus* and *Helicobacter.* These different observations of microbiota responses can be attributed to physicochemical characteristics in the lumen and mucosa including oxygen and nutrient availability (Van den Abbeele et al., 2011; Albenberg et al., 2014; Friedman et al., 2018).

## 4 Mucosa-associated microbiota and intestinal health

**Fig. 1:**
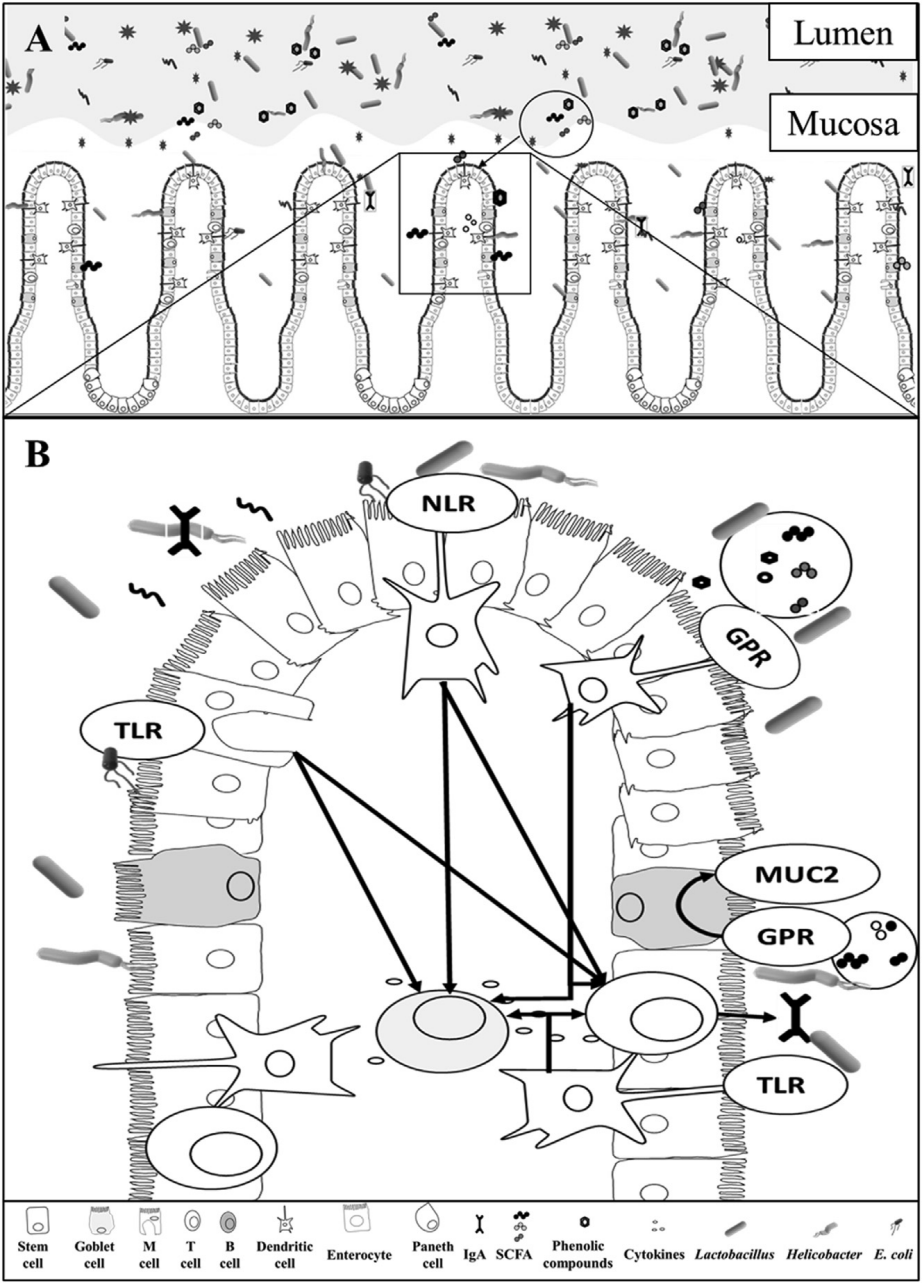
The distinct microbiota profile in the intestinal lumen and mucosa and its interaction with the immune system (Adhikari et al., 2019; Burrough et al., 2017; Mu et al., 2017; De Rodas et al., 2018), drawn by Marcos E. Duarte. (A) The luminal microbiota interacts with digesta, affecting nutrient digestion in addition to secretion of metabolites that would further affect the immune response. (B) Mucosa-associated microbiota directly crosstalk with intestinal immune cells (Arpaia et al., 2013; Belkaid and Hand, 2014; Mulder et al., 2011). Epithelial cells, M cells, and dendritic cells directly sense mucosa-associated microbiota and metabolites inducing the production of Toll-like receptors (TLR), and nucleotide-binding oligomerization domain-like receptors to recruit both T and B cells to aid in the production of cytokines and immunoglobulins (Gutzeit et al., 2014). Toll-like receptors recognize the lipopolysaccharides (LPS) in the cell wall of Gram-negative bacteria inducing the production of nuclear factor kappa β (NF-κβ), tumor necrosis factor-alpha (TNF-α), and interleukin 8 (IL-8) (Stephens and von der Weid, 2020). Goblet cells proliferation are upregulated directly by the mucosa-associated microbiota or by the short-chain fatty acids (SCFA) increasing the production of mucin 2 (MUC2) (Zhang et al., 2017). Dendritic cells recognize metabolites, including SCFA and directly activate G protein-coupled receptors (GPR) recruiting immune cells (Sina et al., 2009) inducing the intestinal immunoglobulin A (IgA) production and reducing the expression of C-X-C motif chemokine ligand 8 (CXCL8) or (IL-8), C–C motif chemokine ligand 20 (CCL20), TNF-α, and interferon gamma (IFN-γ) (Diao et al., 2019; Wen et al., 2012; Zhong et al., 2019). The production of IgA prevents the translocation of bacteria through the epithelial layer and contribute to the modulation of the mucosa-associated microbiota.

The mucosa-associated microbiota can directly affect the intestinal health of pigs utilizing different mechanisms of interaction with enterocytes of host animals. A proposed interaction between intestinal microbiota and the intestinal health is illustrated in Fig. 1.

### 4.1 Interaction between microbiota and epithelial cell receptors

Most of the genes that have been shown to influence the mucosa-associated microbiota are related to the immune system, suggesting that the immune system exerts selective pressure on the intestinal microbiota to promote favorable communities (Honda and Littman, 2016). Whereas, the mucosa-associated microbiota greatly contribute to modulation of the intestinal immune function (Leshem et al., 2020; Paone and Cani, 2020). The intestinal mucosa is composed of epithelial cells, gut-associated lymphoid tissue (GALT), and the mucus layer. The mucosa-associated microbiota, intestinal epithelial cells, and intestinal immune cells engage in complex crosstalk (Garrett et al., 2010; Ma et al., 2018) forming a dynamic and delicate interaction that is critically important for the nutritional and immune function of the intestinal tract. Epithelial cells, M cells, and dendritic cells can directly sense intestinal antigens, inducing the production of Toll-like receptors (TLR), and nucleotide-binding oligomerization domain-like receptors to recruit both T and B cells to aid in the immune response (Gutzeit et al., 2014) (Fig. 1). Cytokines, chemokines, along with host and microbial metabolites are key molecular mediators of intestinal homeostasis that influences the response of both the host and microbiota (Ma et al., 2018). In addition, Sinkora et al. (2002) reported that the occurrence of both T and B cells markedly depends on the interaction of the immune system with the microbiota.

### 4.2 Microbiota metabolites

The cell wall compounds and the ability of the microbiota to adhere to the intestinal epithelial cells interacts with receptors in the epithelial cells affecting the intestinal immune response (Donaldson et al., 2015; Stephens and von der Weid, 2020). Metabolites produced from microbial fermentation and proliferation also play an important role for intestinal microbiota affecting the intestinal immune system (Arpaia et al., 2013; Fan et al., 2015; Feng et al., 2018a; Levy et al., 2016; Rooj et al., 2010). These functions can modulate the immune system toward health or disease, depending on the balance of the intestinal microbiota (Jacobs and Braun, 2015; Perez-Lopez et al., 2016).

Short-chain fatty acids (SCFA) are the major microbial metabolites produced from carbohydrates and the carbon chain of amino acids (AA) (Macfarlane et al., 1992; Nakatani et al., 2018). Bacteria from the phylum Bacteroidetes are well known for their ability to produce acetate and propionate and bacteria in the Firmicutes are efficient butyrate producers (Høverstad and Midtvedt, 1986; Macfarlane and Macfarlane, 2003). However, SCFA production depends on the substrate availability and microbiota composition (Holmes et al., 2020). Some bacteria also produce lactate and succinate, which can be absorbed by the intestinal cells or further converted to propionate by the microbiota (Kajihara et al., 2017).

Besides the contribution to energetic metabolism, SCFA can exert beneficial effects on the intestinal immune system (Venegas et al., 2019). The SCFA can directly activate G protein-coupled receptor 43 and 109A (GPR43 and GPR109A) in neutrophils, macrophages, and dendritic cells (Yang et al., 2018). G protein-coupled receptor 43 is essential for the recruitment of immune cells during intestinal inflammation (Sina et al., 2009). Wu et al. (2017) reported that acetate induced intestinal IgA production mediated by GPR43. Whereas, Iraporda et al. (2015) evaluating the flagellin stimulation on Caucasian colon adenocarcinoma (Caco-2) cells, reported that butyrate and propionate reduced the expression of C-X-C motif chemokine ligand 8 (CXCL8; interleukin 8 [IL-8]) and C–C motif chemokine ligand 20 (CCL20). Furthermore, butyric acid has been shown to reduce the concentration of tumor necrosis factor-alpha (TNF-α) and interferon-gamma (IFN-γ) in the intestine of nursery pigs (Diao et al., 2019; Wen et al., 2012; Zhong et al., 2019). The SCFA are also important in cell proliferation (Cucchi et al., 2020), epithelial barrier function (Diao et al., 2019), and production of important factors for host defense (Fig. 1).

Before digestion and absorption, the protein in the lumen can be fermented along the intestine producing a range of metabolites that can affect the immune system. The products of AA fermentation include SCFA and branched-chain fatty acids (BCFA), ammonia, amines, hydrogen sulfide, phenols, and indoles (Macfarlane et al., 1992; Pieper et al., 2016). These compounds have been related to being either deleterious or beneficial to intestinal health (Fan et al., 2015). The salicylic acid and α-ketoglutaric acid produced by *F. prausnitzii* possess anti-inflammatory effects that can block nuclear factor-κβ (NF-κβ) activation and IL-8 production (Miquel et al., 2015; Sokol et al., 2008).

### 4.3 Microbiota cell wall components

Receptors located on intestinal cells can identify the cell wall structures of mucosa-associated and activate the immune response (Huntley et al., 2018; Royet et al., 2011; Wolf and Underhill, 2018). Lipopolysaccharides (LPS), found in the outer membrane of Gram-negative bacteria are well known for their immunogenicity properties (Roh et al., 2015; Ruemmele et al., 2002) and deleterious effect on tight junction proteins (Nighot et al., 2017). Toll-like receptor 4 (TLR4) and cluster of differentiation 14 (CD14) are receptors present in epithelial cells that recognize the LPS, inducing the NF-κβ, TNF-α, and IL-8 (Stephens and von der Weid, 2020). Another cell wall substance found in the microbial cell wall is peptidoglycan (PG), a potential immunopotentiator that can reduce the inflammatory response and increase humoral immunity (Sasaki et al., 1987; Wolf and Underhill, 2018). The peptidoglycan recognition proteins (PGLYRP1–PGLYRP4) bind to peptidoglycans in microbial cell walls resulting in antibacterial activity (Royet et al., 2011). Peptidoglycans are also important to the proper development of the immune system (Wolf and Underhill, 2018). Ha et al. (2006) reported that PG induces the production of IgA by pattern-recognition receptors (PRR) on the innate intestinal epithelium in mice. The IgA is secreted into the lumen, limiting bacterial colonization and preventing penetration of bacteria through the epithelial layer (Benveniste et al., 1971; Brandtzaeg, 2007; Macpherson et al., 2005; Rios et al., 2016) as shown in Fig. 1.

Some bacteria possess cell membrane adhesins that have been shown to directly promote the immune response of the host (Van Den Broeck et al., 2000; Devriendt et al., 2010; Wang et al., 2019b). Before colonization or infection, the microorganisms adhere to the epithelial cells facilitated by fimbrial or no-fimbrial adhesins. The most common adhesin mechanism in the swine industry are the fimbria F4 and F18 expressed in enterotoxigenic *Escherichia coli* (ETEC) (Dubreuil et al., 2016; Li et al., 2020b; Luise et al., 2019b; Rhouma et al., 2017) as shown in Fig. 1. These structures are related to the virulence factors of ETEC (Fr¨mmel et al., 2013; Kaper et al., 2004; Karch et al., 1985)*.*

## 5 Interaction among diets, microbiota, and immune system

The composition and the abundance of both luminal and mucosa-associated microbiota are largely affected by dietary factors. Intestinal immune system would be influenced by dietary factor directly or indirectly related to the composition and the abundance of intestinal microbiota. Influence of major nutrients and feed additives to intestinal microbiota and immune systems is reviewed in this section.

### 5.1 Interaction among dietary protein, intestinal microbiota, and immune system

Diet is the most important factor affecting the intestinal microbiota of pigs from lactation through the finishing phase. Most of the dietary proteins are digested and absorbed in the small intestine, whereas the undigested protein reaches the large intestine and is fermented by the microbiota. It is important to note that microbiota in the small intestine also have the ability to ferment proteins however, to a lower extent (Davila et al., 2013). The level of crude protein in the diet can affect the intestinal microbiota by increasing the nitrogen availability as well as the pH of the digesta, favoring the proliferation of proteolytic bacteria and potential pathogens (Kim et al., 2018). The major bacteria fermenting protein in the small intestine include *Klebsitella* spp., *E. coli, Streptococcus* spp.*, Succinivibrio dextrinosolvens, Mitsuokella* spp.*,* and *Anaerovibrio lipolytica* (Dai et al., 2010). However, in the large intestine of monogastric animals, the proteolytic activity has been mainly attributed to the genera of *Bacteroides, Propionibacterium, Streptococcus, Fusobacterium, Clostridium,* and *Lactobacillus* (Davila et al., 2013)*.* Interestingly, according to Chen et al. (2018b)*,* reducing the dietary crude protein from 18% to 15% decreased the abundance of harmful bacteria including *Streptococcus* and increased those considered beneficial, *Lactobacillus and Bifidobacterium*, in ileal digesta of growing pigs. Besides the modulation of the microbiota, the CP level can affect the profile of metabolites produced during fermentation (Fan et al., 2015; Chen et al., 2018b). Protein fermentation in the intestine produces AA, SCFA, BCFA, and polyamines that are known to affect intestinal health. In addition, increasing protein fermentation in the small intestine may affect their availability for absorption by the host, thereby reducing the digestibility of AA.

The use of highly digestible protein supplements may reduce the availability of protein for microbial fermentation which can modulate the intestinal microbiota. Iakhno et al. (2020) reported that the use of yeast, replacing 40% of crude protein in the diet, reshaped the microbiota in the ileal and colonic digesta of nursery pigs. Ortman et al. (2020) reported that different protein supplements modulated the microbiota in ileal digesta of weaned pigs, which can be related to the antinutritional factors in the ingredients. Additionally, Wen et al. (2018) reported that the microbial metabolites in the large intestine were affected by different protein sources, whereas it was correlated with the crude protein level within the diet.

The availability of AA can also affect microbiota fermentation. Tryptophan can be metabolized by the intestinal microbiota producing indole-3-acetic acid (IAA) which are ligands for the aryl hydrocarbon receptor (AHR). Aryl hydrocarbon receptor induces the expression of interleukin 22 (IL-22) by the intestinal immune cells (Lamas et al., 2016), further inhibiting intestinal inflammation and enhancing barrier function (Parks et al., 2016).

### 5.2 Interaction among dietary fiber, intestinal microbiota, and immune system

Dietary fiber is one of the dietary compounds most related to intestinal microbiota. After weaning the fiber content in the diet is one important antinutritional factor (ANF) affecting the health of pigs. The ANF effect of fiber is greater in nursery pigs due to the immature intestine being unable to handle the fiber properly. Pigs do not endogenously produce enzymes capable of degrading non-starch polysaccharide (NSP), however, the intestinal microbiota have a broad range of various enzymes related to NSP hydrolysis (Wang et al., 2019c). The soluble portion of the NSP can increase the viscosity of digesta, decreasing the digestibility and altering the environment in the intestinal lumen (Duarte et al., 2019). This change can affect the passage rate, nutrient availability, and oxygen diffusion creating a propitious environment for potential pathogens (Jha et al., 2019). Moreover, Nguyen et al. (2020) proposed that the size of the NSP polymer affects the microbiota as *Bifidobacterium longum, Prevotella copri, Bacteroides plebeius,* and *Bacteroides* sp. primarily utilize soluble arabinoxylan producing oligosaccharides and SCFA, and lactate. Whereas *Subdoligranulum* sp., and *Blautia obeum* mostly utilize oligosaccharides.

Fiber can be utilized by the intestinal microbiota in cross-feeding or a cell-dependent action. This may indicate that supplementation of a single enzyme may affect those bacteria utilizing the target subtract and the oligosaccharides released (Feng et al., 2018b). Moreover, the AXOS release by enzymes would affect both luminal and mucosa-associated microbiota differently from those directly supplemented (Petry et al., 2021). Therefore, feed formulation is an important tool in manipulating the intestinal microbiota to promote the health and performance of pigs.

### 5.3 Interaction among feed additives, intestinal microbiota, and immune system

Selected feed additives directly or indirectly influence the composition and the abundance of both luminal and mucosa-associated microbiota, which in turn influence intestinal immune system. The effects of dietary intervention on the modulation of intestinal microbiota and health of pigs are summarized in Table 1.

**Table 1:** Dietary intervention on modulation of intestinal microbiota and health in pigs.

Initial BW, kg	Days fed	Feed intervention	Site of sampling	Microbiota change	Health and growth performance	Reference
7.7	21	*Bacillus subtilis*	Cecal lumen	↓Enterobacteraceae	Increased immunocompetence, and AA metabolism	Luise et al. (2019a)
6.3	27	*B. subtilis* (ETEC challenged)	Fecal	↓Bacteriodetes, Proteobacteria, and Firmicutes:Bacteroidetes ratio	Reduced *Escherichia coli* shedding and enhanced intestinal integrity	Brooks (2017)
6.3	42	*B. subtills and Bacillus licheniformis*	Jejunal mucosa	↓Cyanobacteria	Increased growth performance, reduced fecal score, and enhanced intestinal integrity	Brooks (2017)
			Jejunal digesta	No effect		
7.1	15	*B. subtills* and *B. licheniformis* (ETEC challenged)	Colonic mucosa	↑*Clostridium, Lactobacillus,* and *Turicibacter*	Increased expression of Atoh1 and ileal goblet cells	Zhang et al. (2017)
7.6	28	*B. subtills* and *Bacillus pumilus* (ETEC challenged)	Jejunal lumen	↓Lachnospiraceae, Ruminococcaceae, Atopobiaceae, Bifidobacteriaceae, Desulfovibrionaceae, Pasteurellaceae	Enhanced growth performance, increased expression of MUC2 and reduced PTGS2 and IL-1β. Reduced the percentage of lymphocytes and increased the percentage of neutrophil in blood Reduced fecal score	He et al. (2020a)
			Ileal lumen	↓Erysipelotrichaceae, Lachnospiraceae, Lachnospiraceae, Atopobiaceae, Bifidobacteriaceae		
			Colonic lumen	↓Atopobiaceae		
4.9	NA	*B. licheniformis* and *Saccharomyces cerevisiae* (ETEC challenged)	Cecal lumen	↑*Lactobacillus*, ↓*E. coli*	Increased growth performance and IgA in jejunal and ileal mucosa; reduced intestinal permeability	Pan et al. (2017)
7.7	16	*Bifidobacterium longum* and *Bifidobacterium animalis* (*Salmonella* challenged)	Fecal	↓*Salmonella typhimurium*	Reduced fecal score, increased acetic acid production, villus height and crypt depth in ileum.	Barba-Vidal et al. (2017)
8.2	21	*Lactobacillus plantarum*	Colonic lumen	↑Prevotellaceae, Bifidobacteriaceae; ↓Campylobacteraceae, Spirochaetae	Reduced fecal score; enhanced jejunal histomorphology, and the humoral immunity preventing inflammation	Wang et al. (2019d)
NA	28	*L. plantarum*	Fecal	↑Diversity and richness. ↑Lactic acid bacteria. ↓Prevotellaceae; ↑Erysipelotrichaceae, Sphaerochaetaceae, Spirochaetaceae and Christensenellaceae	Increased serum IgG, down-regulated genes related to immune system and enhanced integrity epithelial layers in ileum	Shin et al. (2019)
6.0	10	*S. cerevisiae*	Fecal	↑*Enterococcus, Dorea, Bacteroides, Holdemania, Roseburia, Faecalibacterium,* and *Mitsukella*	Increased growth performance and reduced diarrhea incidence.	Xu et al. (2018)
6.5	21	MOS (ETEC challenged)	Cecal digesta	↑*Lactobacillus, Bifidobacterium,* and *Bacillus,* ↓*E. coli*	Increase IgA, IgG and reduced TNF-α, IL-1β, and IL-6 in serum. Enhanced the small intestine integrity.	Yu et al. (2020)
7.4	28	Xylo-oligosaccharide	Colonic digesta	↓*Lactobacillus,* ↑*Streptococcus* and *Turicibacter*	Enhanced intestinal permeability. Reduced the concentration of IFN-γ in serum	Yin et al. (2019)
6.0	48	Cell wall of *S. cerevisiae* (mycotoxin challenged)	Jejunal mucosa	↑*Prevotella* spp., *Turicibacter sanguinis, Clostridium* sp*.*; ↓*Lactobacillus equicursoris*	Reduced TNF-α, IgA, IgG, and protein carbonyl in jejunal mucosa.	Kim et al. (2019)
NA	35	Cell wall of *S. cerevisiae* (mannan-rich fraction)	Cecal lumen	↑*Paraprevotella;* ↓*Prevotella, Suterella, Campilobacter,* and *Akkermansia* at 7 d postweaning*;* ↑*Clostridium* and *Mitsukella,* ↓*Coprococcus* and *Roseburia* at 21 d postweaning	Increased villus height and gene expression related to cellular development and homeostasis, immune-modulation, and protein synthesis.	Fouhse et al. (2019)
7.0	35	β-mannanase	Ileal and cecal digesta	↓*E. coli* in cecal digesta	Increased fat digestibility and enhanced intestinal integrity.	Jang et al. (2020b)
25.4	46	Low fiber (LF), high fiber (HF), HF + xylanase, HF + AXOS	Ileal mucosa	Xylanase: ↑*Bifidobacterium*, *Megasphaera*, and *Chlamydia.* ↓*Clostridium*, and *Escherichia shigella.* AXOS: ↑*Megasphaera*, *Streptococcus.* ↓Candidatus arthromitus, *Helicobacter*	Increased gene expression of enzymes associated with fiber degradation, pentose metabolism, and SCFA production. Reduced oxidative stress and enhanced intestinal barrier integrity.	(Petry et al., 2020, 2021)
			Ileal lumen	Xylanase: ↑Lachnospiraceae *Actinobacillus, Bifidobacterium*, *Lactobacillus.* ↓*Streptococcus*, *Turicibacter.* AXOS: ↑Lachnospiraceae*.* ↓*Actinobacillus,* Pasteurelaceae.		
7.9	20	Xylanase and *Bacillus* sp. (ETEC challenged)	Jejunal mucosa	↓Diversity and *Campylobacter hyointestinalis*	Reduced fecal score, oxidative stress, enhanced growth performance, immune status, and intestinal integrity.	Duarte et al. (2020)
6.4	21	Cocktail^1^	Ileal mucosa	↓*Helicobacter,* ↑*Lactobacillus,*	Decreased TNF-α, homocysteine and increased growth performance	Lo Verso et al. (2020)
			Ileal lumen	↑*Lactobacillus*		
			Colonic mucosa	↑*Lactobacillus,* ↑*Faecalibacterium*		
			Colonic lumen	↑*Faecalibacterium*		
7.7	25	Fermented rice bran extracts	Jejunal mucosa	↑*Streptococcus*	Increased IgG in serum and enhanced growth performance	Zheng (2018)
7.0	35	Lysophospholipids^2^	Jejunal mucosa	↑Firmicutes:Bacteroidetes ratio	Increased litter weigh, the concentration of IL-8 and the enterocyte proliferation in jejunal mucosa	Jang et al. (2020a)
6.2	48	Whey permeate	Jejunal mucosa	↓Firmicutes:Bacteroidetes ratio; ↑Bifidobacteriaceae and Lactobacilaceae, ↓Enterobacteriaceae and Streptococcaceae	Increased IL-8 and enterocyte proliferation. Enhanced growth performance.	Jang et al. (2021)

AA = amino acid; Atoh1 = atonal BHLH transcription factor 1; ETEC = enterotoxigenic *Escherichia coli*; MOS = mannan-oligosaccharides; AXOS = arabinoxylan-oligosaccharides; MUC2 = mucin 2; PTGS2 = prostaglandin-endoperoxide synthase 2; NA = not available; TNF-α = tumor necrose factor alpha; IFN-γ = interferon gamma; SCFA = short chain fatty acid; IL-1β = interleukin 1 beta; IgA = immunoglobulin A; IgG = immunoglobulin G; IL-6 = interleukin 6; IL-8 = interleukin 8.

1 Cocktail (a blend containing bovine colostrum, cranberry extract, carvacrol, yeast-derived mannans, and β-glucans).

2 Sows were fed diets with 0.05% lysophospholipids during lactation and the microbiota was analyzed on the offspring.

#### 5.3.1 Probiotics

Probiotics has been largely used in the swine industry to promote the healthy growth of pigs. The roles of probiotics are intrinsic and related to the host microbiota including competition for nutrients, adhesion sites on the intestinal mucosa, production of lactic acid, SCFA, and anti-microbial compounds (Abriouel et al., 2011; Barba-Vidal et al., 2019; Valeriano et al., 2017). As consequence, these factors, enhance the intestinal barrier function and modulate the immune system (Duarte et al., 2020; Markowiak and Ślizewska, 2018). Lactic acid and SCFA produced by the probiotics change the microenvironment in the intestinal lumen, favoring the proliferation of beneficial bacteria normally related to lower pH (Dowarah et al., 2018). Conversely, the lower pH and the growth of beneficial bacteria lead to an unfavorable environment for the growth of pathogens (Luise et al., 2019a). Antimicrobials produced by some probiotics also help to reduce the proliferation of pathogens (Barba-Vidal et al., 2017). Moreover, bacteria that utilize the metabolites produced by probiotics can also be affected (Wang et al., 2019c). Additionally, probiotics can affect the immune system which then in turn alters the intestinal microbiota composition (Roselli et al., 2017).

Commensal microbes that show some benefit to the host can be potentially considered probiotics. *Bacillus* spp., *Lactobacillus, Bifidobacterium,* and *Enterococcus* are lactic acid-producing bacteria commonly used in probiotic mixtures due to their characteristics (He et al., 2020b; Pringsulaka et al., 2015; Yang et al., 2015). Shin et al. (2019) reported that *Lactobacillus plantarum* probiotic supplemented to pigs from lactation to 4 wk after weaning increased the microbiota diversity and richness*,* the growth of lactic acid bacteria and relative abundance of Erysipelotrichaceae, Sphaerochaetaceae, Spirochaetaceae and Christensenellaceae*,* whereas it reduced the abundance of Prevotellaceae in fecal samples*.* Although a greater abundance of Prevotellaceae has been associated with a healthy microbiota*,* the authors also reported that *L. plantarum* supplementation increased the concentration of serum immunoglobulin G (IgG), downregulated the expression of genes related to immune system and enhanced the epithelial layers in the ileum (Table 1). Zhang et al. (2017) reported that pigs receiving *Bacillus* probiotics increased the abundance of mucosa-associated *Clostridium, Lactobacillus,* and *Turicibacter* increasing the expression of atonal BHLH transcription factor 1 (Atoh1) upregulating the goblet cells proliferation in the ileum. The greater number of goblet cells increased mucin 2 (MUC2) production preserving the intestinal barrier function. These results indicate that the balance of the intestinal microbiota should be considered when evaluating the probiotic effects on the health of the host. In addition, the changes on the fecal microbiota may not be correlated with the immune modulation on the small intestine (Liu et al., 2019). Yeast has also been successfully used as a probiotic modulating the intestinal microbiota and enhancing the small intestinal health of nursery pigs (Elghandour et al., 2020; Xu et al., 2018; Zhaxi et al., 2020) as shown in Table 1.

The use of probiotics should account for the intestinal microbiota status before supplementation (Barba-Vidal et al., 2018). According to Suez et al. (2018) probiotic supplementation in humans can disturb rather than support the intestinal microbiota recovery back to baseline following antibiotic treatment. There is evidence that the host gene expression and the baseline microbiota can affect the probiotic colonization in the intestinal mucosa in humans (Zmora et al., 2018). These findings may indicate that the role of probiotics in the modulation of the intestinal microbiota is more effective in preventing disease-associated dysbiosis by promoting a healthier microbiota, rather than recovery of the microbiota following disruption. Therefore, the approach in dietary probiotic supplementation should consider both the host characteristics and the baseline intestinal microbiota.

#### 5.3.2 Prebiotics

Increasing evidence has shown that oligosaccharides can shift the intestinal microbiota toward species that play an important role in the immune system. This shift in the intestinal microbiota can affect the profile of metabolites produced along the intestine (Singh et al., 2015). Microbial metabolites produced from oligosaccharide supplementation can affect intestinal cell proliferation (Tian et al., 2018), expression of tight junction proteins (Hansen et al., 2019), mucus layer, and modulate the immune response (Guan et al., 2019). Moreover, oligosaccharides can directly affect the immune system by binding specific carbohydrate receptors on intestinal cells resulting in the alteration of the barrier function and immune response (Hansen et al., 2019).

Mannan-oligosaccharides (MOS) are non-digestive carbohydrates comprised of a mannose chain. Most feed additives containing MOS are derived from *Saccharomyces cerevisiae* (Halas and Nochta, 2012). A proposed mechanism of MOS in its role for promoting intestinal health is specific microbes binding the MOS in the intestine and then being transported out on the feces without binding the host cells and therefore, indirectly affecting the immune system. In addition, MOS can directly affect the immune system by stimulating gene expression related to immune response (Che et al., 2011; Halas and Nochta, 2012). Fouhse et al. (2019) reported that a dietary yeast-derived mannan-rich fraction increased the relative abundance of *Mitsuokella* and decreased the relative abundance of *Coprococcus* and *Roseburia* in the cecal digesta of piglets. Additionally, the authors reported an enhancement in intestinal histomorphology and integrity as well as an increase in the jejunal gene expression patterns toward immune modulation. Browne et al. (2019) demonstrated that the mannan rich fraction from yeast reduced the gene expression of TNF-α and TLR4 in intestinal cells (in vitro) by reducing the adherence of *E. coli.* Mannan-oligosaccharides stimulate both systemic and mucosal immunity and modulate the intestinal microbiota in weaned pigs (Valpotić et al., 2016, 2018). According to Upadrasta et al. (2013), the changes in the fecal microbiota of pigs were induced by MOS in the outer layer of cyder yeast. The change to a more beneficial microbiota can reduce the risk of intestinal infection causing diarrhea. According to Yu et al. (2020), supplemental MOS in the diet of weaned pigs enhanced the intestinal integrity by modulating the microbiota in cecal digesta and reducing the inflammatory response caused by *E. coli* K88^+^ (Table 1).

Xylo-oligosaccharide (XOS), a functional carbohydrate derived from the hydrolysis of xylan, has been demonstrated to modulate the intestinal microbiota and the immune system of the host. Xylo-oligosaccharide has been shown to selectively stimulate the proliferation of bacteria generally associated to promote health benefits to the host (Okazaki et al., 1990). Mäkeläinen et al. (2010) reported that XOS promotes both *Bifidobacterium* and *Lactobacillus* proliferation in vitro. According to Okazaki et al. (1990), the ability to utilize XOS of *Bifidobacterium* and *Lactobacillus* at species level depends on the degree of polymerization of the XOS. Moreover, the authors reported that XOS was not utilized as an energy source by *Staphylococcus*, *E. coli*, and most *Clostridium* species. The microbial ability to ferment XOS varies with the source of XOS (Madhukumar and Muralikrishna, 2012). Pan et al. (2019) reported that the XOS dietary supplementation in grow-finishing pigs increased the abundance of *Lactobacillus, Ruminococcus, Coprococcus,* and *Roseburia* as well as increased the concentration of SCFA whereas reduced the abundance of *E. coli* and *Corynebacterium* and the concentration of 1,7-heptanediamine in colonic digesta. The 1,7-heptanediamine is a bioamine related to the AA decarboxylation, therefore this result may suggest that the XOS supplementation could inhibit decarboxylation of amino acids, probably by reducing proteolytic bacteria. Yin et al. (2019) investigated the effects of dietary XOS on intestinal functions and performance of weaned pigs and concluded that dietary XOS increased the microbial α-diversity and increased the abundance of *Streptococcus* and *Turicibacter*, increased the ZO-1 expression, and reduced the concentration of serum IFN-γ in colonic digesta (Table 1). Additionally, the authors state that the abundance of *Lactobacillus* was reduced without affecting growth performance. The authors also reported that XOS supplementation reduced pentadecanal and increased SCFA, coenzyme Q6, and zizyphine A in the distal intestinal digesta. These compounds are probably produced by the intestinal microbiota and further investigation is needed to evaluate their interaction with the host.

In addition to the effect of dietary XOS on the anti-inflammatory response mediated by the intestinal microbiota, XOS can directly affect the immune system (Singh et al., 2015). Nabarlatz et al. (2007) reported that almond shell XOS showed direct immunomodulatory activity. Moreover, Hansen et al. (2019) suggested that the XOS can improve the intestinal barrier function regardless of the microbiota in rats. However, whether the immune response is related to the modulation of the microbiota or by binding cell receptors directly is not clear.

#### 5.3.3 Postbiotics

As discussed above, most of the health benefits associated with prebiotic, probiotic, and synbiotic supplementation is related to the interaction of the microbial metabolites with intestinal microbiota and host cells. Fermentate is a term used in the food industry to describe the product derived from fermentation process containing microorganisms, non-viable cells of fermenting microorganisms, culture medium, fermented substrates, and metabolites (Mathur et al., 2020). Fermentates and microbial extracts including non-viable cells, bioamines, SCFA, cell wall structures, and compounds produced through fermentation by probiotics that promote health effects are known as postbiotics (Wegh et al., 2019), a relatively new term in the animal feed industry. Yeast culture (Mathew et al., 1998; Shen et al., 2009), yeast cell wall extracts (Holanda et al., 2020; Kim et al., 2019), and lactic acid bacteria fermentates (Casey et al., 2007; Mathur et al., 2020) are the traditional postbiotics used in pig production. One mechanism of postbiotic action in the host immune system was proposed in an in vitro study by Wang et al. (2013). The authors reported that heat-treated *Lactobacillus casei* increased the transcription of TLR. The production of TLR drives both T and B cells response leading to IgA production (Gutzeit et al., 2014; Pabst and Slack, 2020). A *Bifidobacterium*-based postbiotic has been shown to further reduce inflammation in intestinal cells by reducing the secretion of IL-8 in an in vitro study (Imaoka et al., 2008). The peptidoglycan present in the cell wall of both Gram-positive and Gram-negative bacteria also play important roles in the interaction between the immune system and mucosa-associated microbiota (Wolf and Underhill, 2018).

According to Xiong et al. (2015), a postbiotic from *S. cerevisiae* fermentate and hydrolyzed cell wall from *S. cerevisiae* increased IgA level in the duodenal and ileal mucosa of weaned pigs. Shen et al. (2009) reported that the use of yeast culture reduced the *E. coli* counts in cecal digesta and reduced the IFN-γ level in the jejunum of nursery pigs. Conversely, Kim et al. (2019) reported that postbiotic yeast cell wall-based reduced IgA and the abundance of pathogenic bacteria in jejunal mucosa of nursery pigs (Table 1). The aforementioned studies show conflicting results on the effects of yeast-based postbiotics on the immune response of pigs and thus further investigation considering the interaction among postbiotic, mucosa-associated microbiota, and immune system is required.

#### 5.3.4 Enzymes

The effect of enzymes on the intestinal microbiota is related to the changes in the physicochemical properties of the substrate in the intestinal lumen and the release of prebiotics, and bioactive compounds (Duarte et al., 2020; Petry et al., 2021). The oligosaccharides released by NSP degrading enzymes (NSPase) can increase the fermentability of the dietary fiber by the intestinal microbiota thereby increasing SCFA production in the intestine (Den Besten et al., 2013; Nakatani et al., 2018). Xylanase, β-glucanase, and β-mannanase are examples of NSPase largely incorporated in animal feed (Kiarie et al., 2013). The use of xylanase hydrolyzing xylan has been reported to reduce the digesta viscosity (Chen et al., 2020; Duarte et al., 2019) which can alter the physicochemical characteristics of the luminal content and increase the nutrient availability for host utilization (Zhang et al., 2018b). The environmental change in the intestinal lumen and the released oligosaccharides can selectively increase the abundance of fiber-degrading bacteria rather than proteolytic bacteria (Akkerman et al., 2020; Munyaka et al., 2016; Zhang et al., 2018b). Petry et al. (2021) reported that dietary xylanase supplementation modulated the mucosa-associated microbiota in pigs fed corn-based arabinoxylans (Table 1). The authors also reported that xylanase supplementation increased the expression of genes related to enzymes-degrading arabinoxylan and AXOS, ferulic acid esterase, and production of butyric acid in the ileal mucosa. Commercially available xylanase may also contain feruloyl esterase produced by the microorganisms-producing xylanase (Petry and Patience, 2020) that release phenolic compounds cross-linked to xylan (Mafa et al., 2021; Mathew and Abraham, 2004; Mkabayi et al., 2020). Ferulic acid, the major phenolic compound in the plant cell wall is a potent antioxidant that can directly affect the host antioxidant status (Wang et al., 2020). Studies have shown that ferulic acid possesses antibiotic properties (Borges et al., 2013) that can modulate the intestinal microbiota by reducing ETEC K88 and F18^+^ growth in porcine feces (Arzola-Alvarez et al., 2020). Beta-Mannanase also plays a role in the intestine of pigs by reducing the immune response and modulating the intestinal microbiota (Kiarie et al., 2016). According to Jang et al. (2020), β-mannanase supplementation reduced the count of *E. coli* in cecal digesta and enhanced the jejunal histomorphology of weaning pigs.

The role of phytase on the intestinal microbiota may be related to buffering property the availability of Ca and P for microbial fermentation (Bovee-Oudenhoven et al., 1997). Studies have been reported that P and Ca levels can modulate the gastrointestinal microbiota, increasing the abundance of beneficial bacteria and reducing potential pathogens counts (Metzler-Zebeli et al., 2013). Mann et al. (2014) reported that the greater levels of available Ca and P in the diet of nursery pigs modulated the mucosa-associated microbiota by increasing the abundance of *Lactobacillus* in stomach mucosa, *Citrobacter freundii* in ileal mucosa, and *P. copri* in colonic mucosa. Phytic acid reduces the P and Ca availability for the host as well as microbiota metabolism (Heyer et al., 2019). Microbiota compete with the host for the available P although most intestinal microbiota can express phytase when the available P level is critically low (Dersjant-Li et al., 2015). Therefore, adding phytase to the diet would provide P and Ca for the host and microbiota metabolism. According to Klinsoda et al. (2020), dietary phytase can shift the microbiota along the digesta mucosa-lymph node axis in the ileum of nursery pigs. Moreover, Metzler-Zebeli et al. (2020) reported that dietary supplementation with phytase increased the abundance of Clostridiaceae and Ruminococcaceae in the feces of growing to finish pigs.

Supplementation of multi-enzymes may show a synergetic effect due to the complexity of plant cell wall components and therefore the variety of oligosaccharide and bio-compounds released. Li et al. (2020a) reported that a multi-carbohydrase complex containing xylanase, β-glucanase, and pectinase recovered the intestinal microbiota homeostasis disrupted by ETEC challenge in ileal and colonic digesta of newly weaned pigs. Kim et al. (2018) reported that the inclusion of multi-enzymes containing xylanase, amylase, β-mannanase, protease, and phytase increased the count of *Lactobacillus* spp. and decreased of *E. coli* and *Clostridium* spp. in digesta of ileum and cecum.

#### 5.3.5 Phytobiotics

A broad range of plant extracts including essential oils, phenolic compounds, and resins have been used in the animal industry as probiotics (Mohammadi Gheisar and Kim, 2018). Phytobiotics, also known as phytogenic feed additives (PFA), have been used in feed to promote growth performance by enhancing intestinal health and modulating the intestinal microbiota (Blavi et al., 2016; Clouard and Val-Laillet, 2014; Kroismayr et al., 2008; Liu et al., 2013; Modina et al., 2019; Windisch et al., 2008).

Essential oils (EO) are extracts derived from plants that have been used as phytobiotics to promote the health and growth performance of livestock due to their properties including antimicrobial capacity (Omonijo et al., 2018). The proposed antimicrobial mechanism of EO is related to the alteration of the cell wall and cytoplasmic membrane, increasing the cell permeability and reducing the virulence function (Nazzaro et al., 2013). Man et al. (2019) analyzed (in vitro) the inhibitory and bactericidal activity of various EO against *Staphylococcus aureus, Enterococcus faecalis, E. coli, Klebsiella pneumoniae* and *Pseudomonas aeruginosa*. The authors concluded that the most active EO were oregano, thyme, and lemon oil because of the great concentration of terpenes and terpenoids in these oils. Cheng et al. (2018) reported that oregano essential oil (OEO) reduced *E. coli* counts in the ileal digesta and improved intestinal morphology, the antioxidative capacity, and growth performance of growing-finishing pigs. Furthermore, the offspring of sows fed diets containing OEO during late gestation and lactation had improved growth performance and health due to the modulation of the fecal microbiota (Hall et al., 2021). Piglets from sows fed diets with OEO increased the abundance of Spirochaetaceae*,* Peptostreptococcaceae, Ruminococcaceae, Erysipelotrichacea, and Lachnospiraceae in feces (Table 1).

Cardol and anacardic acid from cashew nutshell have shown antimicrobial activity against both Gram-positive and Gram-negative bacteria (Hollands et al., 2016). Cardol and anacardic acid are potential protonophores and ionophores (Toyomizu et al., 2003) that can cause damage to the cell membrane of bacteria (Abbas et al., 2012). Moreover, anacardic acid can induce neutrophil extracellular trap production by neutrophils that can facilitate the entrapment and killing of bacteria (Hollands et al., 2016). Therefore, cashew nutshell products can modulate the intestinal microbiota by directly killing bacteria or by modulating the host immune system that will further interact with the microbiota. Moita et al. (2021) showed that increasing supplementation of cashew nutshell products improved the intestinal health and the composition of mucosa-associated microbiota in the jejunum of nursery pigs by reducing the relative abundance of Helicobacteraceae, whereas increasing *Lactobacillus kitasatonis*.

Collectively, the effect of the numerous feed additives promoting health and growth response in pigs can be associated with the changes in the intestinal microbiota. Considering the microbiota modulation, the properties of the feed additives can be characterized by antimicrobial activity and by feeding selected microbiota. Therefore, the use of feed additives to modulate the microbiota at a specific level should consider the existing microbial community before nutritional interventions are put in place in order to promote a more precise response.

## 6 Conclusion

Modulation of intestinal microbiota toward a more beneficial microbial community can be a key factor in enhancing intestinal health and therefore increasing the growth performance of nursery pigs. The intestinal microbiota in the lumen and mucosa play an important role along the entire length of the intestine. The role of the microbiota in the lumen is more related to the digestive function, producing metabolites that further can interact with the host. Whereas, the mucosa-associated microbiota directly interacts with the epithelial cells in the intestine by using the adherence system and by producing metabolites directly secreted on the intestinal cells. The mucosa-associated microbiota also regulates the mucus production, a physical barrier against pathogenic adherence. *Prevotella, Lactobacillus,* and *Bifidobacterium* have great abundance in the mucosa and are associated with health benefits. Whereas, *Campylobacter, Clostridium, Veillonella,* and *Helicobacter* are potentially harmful or associated with intestinal dysbiosis. These bacteria could be used as a biomarker to predict responsiveness to dietary interventions and more specific nutritional intervention depending on genetics, on-farm management, and current nutritional management. Therefore, understanding the roles of intestinal microbiota and their interaction with the host is essential in feed formulation and dietary supplementation in the swine industry.

## Author contributions

**Marcos EliasDuarte**: Methodology; Resources; Software; Validation; Visualization; Roles/Writing – review & editing; **Sung Woo** **Kim**: Conceptualization; Data curation; Funding acquisition; Investigation; Methodology; Project administration; Resources; Software; Supervision; Validation; Roles/Writing – original draft; Writing – review & editing.

## Declaration of competing interest

We declare that we have no financial and personal relationships with other people or organizations that can inappropriately influence our work, and there is no professional or other personal interest of any nature or kind in any product, service and/or company that could be construed as influencing the content of this paper.
